# Acute Aerobic Exercise Remodels the Adipose Tissue Progenitor Cell Phenotype in Obese Adults

**DOI:** 10.3389/fphys.2020.00903

**Published:** 2020-07-28

**Authors:** Alison C. Ludzki, Emily M. Krueger, Toree C. Baldwin, Michael W. Schleh, Cara E. Porsche, Benjamin J. Ryan, Lindsey A. Muir, Kanakadurga Singer, Carey N. Lumeng, Jeffrey F. Horowitz

**Affiliations:** ^1^Substrate Metabolism Laboratory, School of Kinesiology, University of Michigan, Ann Arbor, MI, United States; ^2^Department of Pediatrics, Medical School, University of Michigan, Ann Arbor, MI, United States; ^3^Graduate Program in Immunology, Medical School, University of Michigan, Ann Arbor, MI, United States; ^4^Department of Computational Medicine and Bioinformatics, Medical School, University of Michigan, Ann Arbor, MI, United States; ^5^Department of Molecular and Integrative Physiology, Medical School, University of Michigan, Ann Arbor, MI, United States

**Keywords:** preadipocytes, exercise, obesity, macrophages, endothelial cells, T cells

## Abstract

Adipose tissue pathology in obese patients often features impaired adipogenesis, angiogenesis, and chronic low-grade inflammation, all of which are regulated in large part by adipose tissue stromal vascular cells [SVC; i.e., non-adipocyte cells within adipose tissue including preadipocytes, endothelial cells (ECs), and immune cells]. Exercise is known to increase subcutaneous adipose tissue lipolysis, but the impact of exercise on SVCs in adipose tissue has not been explored. The purpose of this study was to assess the effects of a session of exercise on preadipocyte, EC, macrophage, and T cell content in human subcutaneous adipose tissue. We collected abdominal subcutaneous adipose tissue samples from 10 obese adults (BMI 33 ± 3 kg/m^2^, body fat 41 ± 7%) 12 h after a 60 min acute session of endurance exercise (80 ± 3%HR_peak_) vs. no acute exercise session. SVCs were isolated by collagenase digestion and stained for flow cytometry. We found that acute exercise reduced preadipocyte content (38 ± 7 vs. 30 ± 13%SVC; *p* = 0.04). The reduction was driven by a decrease in CD34^hi^ preadipocytes (18 ± 5 vs. 13 ± 6%SVC; *p* = 0.002), a subset of preadipocytes that generates high lipolytic rate adipocytes *ex vivo*. Acute exercise did not alter EC content. Acute exercise also did not change total immune cell, macrophage, or T cell content, and future work should assess the effects of exercise on subpopulations of these cells. We conclude that exercise may rapidly regulate the subcutaneous adipose tissue preadipocyte pool in ways that may help attenuate the high lipolytic rates that are commonly found in obesity.

## Introduction

Many obesity-related metabolic health complications stem from abnormalities within adipose tissue, including excessive rates of fatty acid release into the systemic circulation and chronic low-grade inflammation. Adipocytes are the primary site for lipid storage in humans and they constitute most of the volume of adipose tissue, but adipocytes represent only ~20% of cells within the tissue (Lee et al., 2013; Raajendiran et al., 2016). The vast majority of these other cell types are found in the stromal vascular fraction of adipose tissue. Adipose tissue stromal vascular cell (SVC) composition is heterogeneous and includes adipocyte precursor cells (preadipocytes), immune cells [e.g., macrophages, T cells, and dendritic cells (DCs)], and endothelial cells (ECs), among others. These cell populations all play a key role in regulating the overall “metabolic health” of adipose tissue. For example, impairments in the ability of these cells to (i) differentiate into new adipocytes, (ii) maintain an anti-inflammatory immune environment, and (iii) propagate angiogenesis to maintain sufficient tissue oxygenation and tissue crosstalk are some of the main maladaptive features commonly found in adipose tissue from obese, insulin resistant adults (Virtue and Vidal-Puig, 2010).

Preadipocytes are self-renewing SVCs that can differentiate into mature adipocytes. During periods of growth/weight gain, these cells can differentiate into new adipocytes in response to adipogenic stimuli including insulin, fatty acids, catecholamines, and cortisol (Ghaben and Scherer, 2019). While the contribution of adipogenesis to mature adipocytes in obesity is relatively low at ~8% new cells per year (Strawford et al., 2004; Spalding et al., 2008), there is evidence that adipogenic rate can be altered/accelerated *in vivo* (White and Ravussin, 2019). Increased adipogenesis may be beneficial during episodes of weight gain by increasing the number of smaller fat cells that sequester bioactive fatty acids as triglycerides. Furthermore, preadipocyte subtypes that differentiate into adipocytes with distinct metabolic phenotypes have recently been identified (Min et al., 2019; Raajendiran et al., 2019). Raajendiran et al. (2019) isolated three distinct preadipocyte populations based on the abundance of CD34 (CD34^−^, CD34^lo^, and CD34^hi^) and reported these preadipocyte subpopulations to feature unique metabolic properties *ex vivo*. Notably, CD34^hi^ preadipocytes differentiate into mature adipocytes with marked metabolic abnormalities, and CD34^hi^ preadipocytes are also more prevalent in adipose SVCs from people with diabetes compared with non-diabetic controls (Raajendiran et al., 2019). Therefore, modifications to the abundance of these different preadipocyte subtypes may impact adipose tissue function and metabolic health.

Insufficient adipose tissue angiogenesis in expanding adipose tissue in obesity is thought to result in adipocyte hypoxia (Sun et al., 2013). ECs are the primary component of adipose tissue vasculature, and EC migration to the tip of capillary sprouts is a key step in angiogenesis (Eilken and Adams, 2010). Therefore, increasing EC migration and function during periods of weight gain could minimize adipose tissue hypoxia and the ensuing chronic low-grade inflammation.

While “healthy” adipose tissue function is maintained by a variety of anti-inflammatory immune cells (e.g., T regulatory cells and M2-like macrophages), adipose tissue hypoxia, pathogen-associated molecular patterns (e.g., lipopoly-saccharide), and adipocyte death are among the triggers for a shift towards chronic low-grade inflammation in obesity. This environment impairs adipose tissue storage and insulin action by increasing pro-inflammatory cytokine production (de Luca and Olefsky, 2008). In turn, adipose tissue inflammation and insulin resistance can exacerbate the high rates of fatty acid release and cytokine production from obese adipose tissue, contributing to systemic insulin resistance.

Acute exercise triggers changes in adipose tissue that may increase adipogenesis, angiogenesis, and inflammatory signaling (Townsend et al., 2017; Van Pelt et al., 2017), suggesting unique potential for exercise to coordinately regulate the SVC composition of obese adipose tissue. While most of these studies have been performed at the whole tissue level, there is a gap in understanding regarding whether acute exercise remodels adipose tissue SVC composition in obese adults. Previous studies have measured inflammatory signaling changes in adipose tissue samples collected 2–24 h after acute exercise (Macpherson et al., 2015; Fabre et al., 2018), suggesting this may be an important window to capture the remodeling of SVCs. Although visceral adipose tissue is typically considered the predominant site of obesity-related inflammation, this is often a consequence of impairments in subcutaneous adipose tissue storage capacity that result in ectopic lipid deposition in visceral adipose tissue, as well as other tissues such as the liver and skeletal muscle. Therefore, the goal of this study was to quantify changes in preadipocytes, ECs, macrophages, and T cells in abdominal subcutaneous adipose tissue 12 h after a single session of moderate-intensity endurance exercise in obese adults.

## Materials and Methods

### Subjects

Ten obese adults (three men and seven women) participated in the study. We only recruited participants who were regular exercisers (≥3 days/week of 30–60 min moderate/vigorous endurance-type exercise) in order to avoid the confounding effects of a novel exercise stimulus. Participants with elevated blood pressure (>140/90 mmHg), history of cardiovascular or metabolic disease, and regular use of medications known to affect metabolism or inflammation were excluded. All women were pre-menopausal, with regularly occurring menses. The study protocol was approved by the University of Michigan Institutional Review Board.

### Preliminary Testing

Subjects were screened for physical activity status, cardiovascular disease risk, and medical history by questionnaires. Resting blood pressure, weight, height, body composition (DEXA; GE Lunar DPX), and peak oxygen consumption (VO_2_ peak) were also measured during screening. The VO_2_ peak test began with a warm-up at a moderate running speed individualized for each subject, after which the incline of the treadmill was increased every 1–2 min until volitional fatigue. During a separate visit, we performed an oral glucose tolerance test (OGTT) after an overnight fast (12 h). Because all subjects were regular exercisers, to avoid the robust influence of acute exercise on insulin sensitivity, the OGTT was conducted exactly 3 days after their most recent exercise session.

### Experimental Trial

#### General Study Design

All subjects completed two separate experimental trials in a randomized order. The only difference between the two trials was whether participants exercised (EX) or remained sedentary (SED) on the evening before adipose tissue sample collection. Each trial required subjects to visit the laboratory in the evening for their supervised EX session (or SED) and again the next morning for blood and adipose tissue sample collection (see Supplementary Figure 1 and more details below). All participants were required to perform their last regular exercise session exactly 2 days before beginning their experimental trial (3 days before sample collection). On the day of their first evening visit, participants recorded their dietary intake and replicated this diet the day before their second experimental trial.

#### Evening Before Sample Collection

Subjects arrived at the laboratory at approximately 1800 h. After resting quietly for 30 min, participants either (1) performed 60 min moderate-intensity EX at 80 ± 3%HR_peak_ (152 ± 8 bpm) or (2) remained SED by resting quietly in the laboratory. EX was completed on a treadmill, except for one participant who used a cycle ergometer. Fifteen minutes after completing the hour of SED/EX, participants were provided with a standardized meal: 33% daily energy expenditure calculated by the Mifflin St. Jeor equation (Mifflin et al., 1990). The meal was composed of ~55% carbohydrate, 30% fat, and 15% protein. Participants then abstained from exercise, food, and drink (other than water) overnight.

#### Morning of Sample Collection

Participants returned to the laboratory at approximately 0645 h. After resting quietly for 30 min, baseline blood samples were collected by venipuncture. Abdominal subcutaneous adipose tissue samples were subsequently collected under local anesthesia (2% lidocaine without epinephrine) by needle aspiration with a Coleman aspiration needle (Mentor Worldwide LLC, Irvine, CA). After sample collection, subjects were provided with a meal and discharged from the laboratory. Our decision to collect adipose tissue samples the day after a session of exercise was based in part on previous work reporting inflammatory signaling changes in adipose tissue samples collected between 2 and 24 h after exercise (Macpherson et al., 2015; Fabre et al., 2018). We specifically chose to collect the adipose tissue samples 12 h after exercise in order to assess the effects of evening exercise (it is common for exercise to be completed in the evening after work) on changes in adipose tissue collected the next morning, in the basal state (i.e., at rest after an overnight fast).

### Analytical Procedures

#### Adipose Tissue Stromal Vascular Cell Isolation

Two grams of adipose tissue were collected into Fluorescence-Activated Cell Sorting (FACS) buffer [0.5% fatty acid-free bovine serum albumin in Hank’s Balanced Salt Solution (HBSS) with calcium and magnesium] and processed by collagenase digestion for isolation of the SVCs. Large pieces within the aspirated sample were minced by hand with sterile scissors before incubating the samples in 3 mg/ml Collagenase Type I (Worthington cat NC9633623) in FACS buffer (~30 mg/g tissue) while rocking at 37°C for ~45 min until samples became slurry. This slurry was passed through a 100 μm filter to isolate the SVCs. Following centrifugation and red blood cell lysis, SVCs were washed, pelleted, and counted with a hemocytometer. SVCs were then stained for flow cytometry and fixed in 0.1% paraformaldehyde until analysis.

#### Flow Cytometry

SVCs were stained for flow cytometry as described previously (Muir et al., 2018) with minor modifications. Briefly, ~1E6 cells were used for full stain and fluorescence minus one (FMO) controls (controls to identify the signal of a given fluorochrome in the context of the full remaining panel of fluorochromes), and ~2E5 cells were used for single stain/unstained controls. Details regarding cell surface markers and fluorophores used in this study are provided in the Supplementary Table. Sample tubes were incubated in Fc block (for nonspecific binding to Fc receptors of immune cells) for 5 min prior to staining. Cells were incubated in conjugated antibodies (Supplementary Table) for 30 min at 4°C (dark), washed with 2 ml PBS, pelleted, and resuspended in PBS for analysis. Samples were analyzed on the LSR Fortessa at the University of Michigan Flow Cytometry Core. FlowJo® software (Treestar) was used to quantify cell populations from human SVCs, which were gated according to Supplementary Figure 2. After excluding adhered cells, debris, and dead cells, T cells (CD45^+^CD3^+^), macrophages (CD45^+^CD64^+^CD206^+^), and DCs (CD45^+^CD64^−^CD11c^+^) were selected off of CD45^+^ total immune cells. ECs (CD45^−^CD34^+^CD31^+^) and preadipocytes (CD45^−^CD31^−^CD29^+^) were selected from the CD45^−^ non-hematopoietic fraction. All cell populations were normalized to the total number of live cells analyzed. The gating strategy is depicted in Supplementary Figure 2.

#### Blood Measurements

Plasma glucose (Thermo Scientific, Waltham, MA), non-esterified fatty acid (Wako Chemicals USA, Richmond, VA, USA), triacylglyceride (Triacylglyceride reagent; Sigma Aldrich), and total‐ and high-density lipoprotein (HDL; Cholesterol E and HDL-Cholesterol E; Wako Chemicals USA) cholesterol concentrations were measured using commercially available colorimetric assay kits. Plasma insulin concentration was measured with a chemiluminescent immunoassay (Siemens IMMULITE 1000, Flanders NJ). Matsuda insulin sensitivity index (ISI) was calculated as described by Matsuda and DeFronzo (1999).

### Statistics

Paired student’s *t*-tests were performed to assess differences between SED and EX. Two-way repeated measures ANOVAs (repeated for exercise and preadipocyte subtype) were used to assess differences in preadipocyte subpopulations, with Sidak’s multiple comparison test when required. Statistical significance was defined as *p* < 0.05.

## Results

### Baseline Subject Characteristics

Participant characteristics are presented in Table 1. We only enrolled subjects who exercised regularly (4 ± 1 sessions of moderate/vigorous exercise per week) to avoid the confounding influence of a novel exercise task on inflammation. Despite being regular exercisers, all subjects were classified to have impaired glucose regulation/insulin resistance, based on their Matsuda ISI (Radikova et al., 2006), which was measured after abstaining from exercise for 3 days (Table 1).

**Table 1:** Baseline characteristics.

Age (years)	26 ± 5
Body mass (kg)	96 ± 11
BMI (kg/m^2^)	33 ± 3
Percent body fat (DEXA)	41 ± 7
Fat mass (kg)	39 ± 7
Fat free mass (kg)	56 ± 7
VO_2peak_ (L/min)	3.0 ± 0.5
Matsuda ISI	4.1 ± 1.4

*n* = 10. Data are mean ± SD.

### Exercise Effects on Fasting Blood Profile

Fasting plasma concentrations of non-esterified fatty acids, triglycerides, Total-, HDL-, and LDL-cholesterol were not affected by the exercise session performed the previous day (SED vs. EX; Table 2). Similarly, fasting plasma glucose and insulin concentrations were nearly identical between EX and SED (Table 2). There was also no change in circulating concentrations of IL6 or TNFα in EX vs. SED (Table 2).

**Table 2:** Exercise effects on fasting blood profile.

	SED	EX	*p*
NEFA (μM)	344 ± 70	346 ± 71	0.97
Triglycerides (mg/dl)	82 ± 40	66 ± 25	0.30
Total cholesterol (mg/dl)	145 ± 27	137 ± 29	0.10
HDL cholesterol (mg/dl)	36 ± 13	34 ± 9	0.27
LDL cholesterol (mg/dl)	109 ± 26	101 ± 24	0.21
Fasting glucose (mg/dl)	78 ± 10	79 ± 10	0.76
Fasting insulin (μIU/ml)	12 ± 5	11 ± 6	0.90
IL6 (pg/ml)	2.4 ± 0.8	2.5 ± 0.7	0.86
TNFα (pg/ml)	0.9 ± 0.3	0.9 ± 0.3	0.91

NEFA, non-esterified fatty acids; HDL, high density lipoprotein; LDL, low density lipoprotein; IL6, interleukin 6; and TNFα, tumor necrosis factor alpha. *n* = 10. Data are mean ± SD.

### Effects of Exercise on Stromal Cell Number

**Figure 1:**
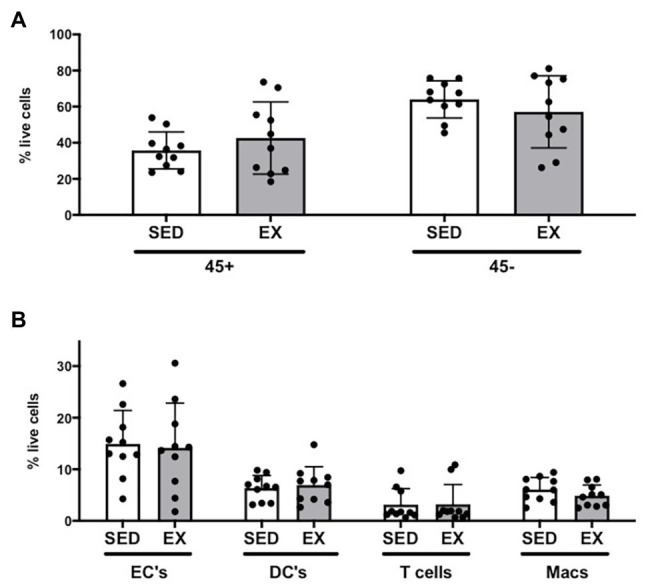
Stromal vascular cell (SVC) content of adipose tissue samples collected during the sedentary (SED) and exercised (EX) visits. **(A)** Effects of exercise on immune cells as CD45^+^ and non-immune cells as CD45^−^; **(B)** effects of exercise on subsets selected from CD45^+^ or CD45^−^ parent populations [endothelial cells (EC), dendritic cells (DC), T cells, and macrophages (Macs)]. All cell populations are represented as %SVC per that sample (% live cells). *N* = 10. Data are mean ± SD. SVC, stromal vascular cells.

**Figure 2:**
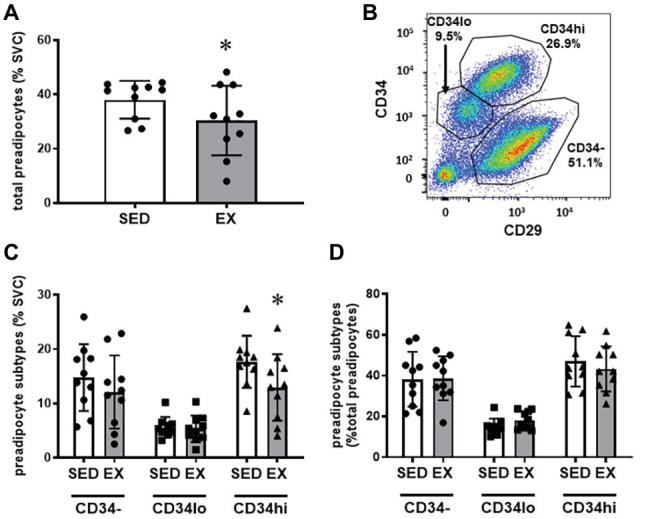
Preadipocyte phenotype. **(A)** Effects of exercise on total preadipocytes in the stromovascular fraction. **(B)** Representative plot showing preadipocyte subtype selection on CD34 and CD29 expression. Full gating strategy is shown in Supplementary Figure 2. **(C)** Effects of exercise on preadipocyte subtypes (CD34^−^, CD34^lo^, and CD34^hi^) expressed as %SVC. **(D)** Effects of exercise on preadipocyte subtypes expressed as %total preadipocytes. *N* = 10. Data are mean ± SD. ^*^p < 0.05 by Sidak’s multiple comparison. SVC, stromal vascular cells.

The exercise session (performed 12 h before adipose tissue collection) did not affect total cell number in the SVC fraction (12.6 ± 3.1 × 10^4^ vs. 12.0 ± 3.9 × 10^4^ total cells for SED and EX, respectively; *p* = 0.74). Exercise also did not affect the proportion of CD45^−^CD31^+^CD34^+^ ECs, total CD45^+^ immune cells, or any of the other immune cell populations we measured (i.e., CD45^+^CD64^−^CD11c^+^ DCs, CD45^+^CD3^+^ T cells, and CD45^+^CD64^+^CD206^+^ macrophages; Figure 1). In contrast, exercise reduced the proportion of total preadipocytes (CD45^−^CD31^−^CD29^+^) in the SVC fraction (*p* = 0.04; Figure 2A).

We stratified preadipocytes into three subtypes according to CD34 and CD29 expression (Figure 2B) as previously performed by Raajendiran et al. (2019). Shown in Figure 2C, these three populations are labeled CD34^hi^ (CD29^+^CD34^hi^), CD34^lo^ (CD29^+^CD34^lo^), and CD34^−^ (CD29^+^CD34^−^). These populations also express variable CD29 expression, with the CD34^−^ population expressing the highest level of CD29 (Figure 2B). Interestingly, the reduction in the preadipocytes in the SVCs appeared to be due to a reduction in the proportion of the CD34^hi^ subtype (Interaction *p* = 0.046; Sidak’s multiple comparison *p* = 0.002), while CD34^−^ and CD34^lo^ preadipocytes were not different in SED vs. EX (Figure 2C). There was no main effect of exercise on preadipocyte subtypes expressed as a proportion of total preadipocytes (*p* = 0.59; Figure 2D).

### Assessing the Presence of Blood Cells in the Stromal Vascular Cell Populations

It was important to establish the influence of blood cells on our SVC quantification, because traces of blood are very common in the adipose tissue samples collected using the lipo-aspiration procedure. In Supplementary Figure 3, panels A–F present whole blood stained with the same antibody panel we used to measure adipose tissue SVCs. As expected, T cells (Supplementary Figure 3B) and DCs (Supplementary Figure 3C) are present in blood; and therefore, the adipose tissue T cell/DC numbers we present above may include blood contamination – though contamination was likely uniform in samples collected at the EX and SED visits. There was no positive EC (Supplementary Figure 3E) or preadipocyte (Supplementary Figure 3F) staining on the whole blood control. Using an anti-CD206 antibody, we were able to identify adipose tissue-specific macrophages. Supplementary Figure 3H shows the CD206 FMO control on SVC’s confirming the presence of CD206^hi^ and CD206^lo^ macrophages. Supplementary Figure 3C shows that there is a large population of CD64^+^ blood cells, a typical macrophage marker. Therefore, we relied on CD206 stratification to exclude the majority of these potential contaminating blood cells (likely monocytes), because Supplementary Figure 3D identifies the vast majority of these cells to be CD206^−^. Supplementary Figure 3H (CD206 FMO on SVCs) shows that the CD206^−^ population of blood contamination was distinct from a population of CD206^lo^ macrophages (which has been previously reported by Wentworth et al., 2010).

## Discussion

This study provides a novel assessment of changes in stromal cell composition in human subcutaneous adipose tissue after a session of endurance exercise. A key finding of this study was that preadipocyte content in the SVC fraction of subcutaneous adipose tissue was reduced 12 h after a session of exercise. Importantly, this reduction in preadipocyte content was driven by a reduction in CD34^hi^ preadipocytes – a population of adipocyte precursor cells that has been linked to the formation of an adipocyte phenotype with elevated lipolytic rate (Raajendiran et al., 2019). In contrast to these exercise-induced changes in preadipocytes, there were no effects of acute exercise on total immune cell number, immune cell subtypes (macrophages, T cells, and DCs), or EC number in subcutaneous adipose tissue. Therefore, these data suggest that a single session of exercise may provide a stimulus to remodel the preadipocyte pool within human subcutaneous adipose tissue, without altering immune cell or EC number 12 h after a single bout of exercise.

Preadipocytes are most often identified by the expression of CD34 (Cawthorn et al., 2012), which is a transmembrane phosphoglycoprotein typically associated with hematopoietic stem/progenitor cells. While the most reliable cell surface markers remain unresolved, the three distinct CD34 subpopulations found in our subjects (CD34^−^, CD34^lo^, and CD34^hi^) were previously reported to differentiate into mature adipocytes with divergent metabolic properties (Raajendiran et al., 2019). In particular, CD34^hi^ preadipocytes differentiate into adipocytes with markedly elevated lipolytic rate, and high rates of lipolysis have been clearly linked with impaired insulin sensitivity (Horowitz et al., 1999; Santomauro et al., 1999). Our finding that exercise lowered the proportion of CD34^hi^ cells in the SVC suggests that the proportion of preadipocyte subtypes in human subcutaneous adipose tissue is plastic, and it is possible that exercising regularly may lead to a shift toward a more favorable preadipocyte phenotype, primed to generate adipose tissue with a lower average lipolytic rate. Alternatively, the reduction in CD34^hi^ preadipocytes may reflect an increased differentiation of this preadipocyte subtype to mature adipocytes. To discern between these two possible outcomes, future studies could assess the expression profile of markers of differentiation and/or cell death in these cell populations. Importantly, meaningful exercise-induced changes likely accrue after repeated exposures to the exercise stimulus, and therefore, exercise training interventions would be required to assess the potential changes in adipocyte phenotype and the subsequent effects on adipose tissue metabolism.

The session of exercise did not affect the relative abundance of CD34^−^ cells, a subset of preadipocytes with high CD29 expression that that has been reported to generate beige-like adipocytes (Xue et al., 2015; Raajendiran et al., 2019). It has been suggested that exercise may induce a beige-like phenotype in white adipose tissue *via* adrenergic signaling and/or myokine release from skeletal muscle (Stanford and Goodyear, 2018). Our finding that exercise did not alter the abundance of CD34^−^ (CD29^hi^) preadipocytes could be interpreted to suggest that the exercise session did not remodel the preadipocyte pool toward beige-like precursors, which is consistent with several studies indicating that exercise training does not induce a beige-like phenotype within subcutaneous adipose tissue in humans (Camera et al., 2010; Stinkens et al., 2018; Tsiloulis et al., 2018).

Adipose tissue is a primary source of circulating inflammatory factors in obesity (Hotamisligil et al., 1993; Kern et al., 1995; Bulló et al., 2002), but the effects of acute exercise on changes in adipose tissue immune cell composition have not been directly measured. Prior methods do not directly quantify adipose tissue macrophage numbers and show discrepant responses to acute exercise (Macpherson et al., 2015; Fabre et al., 2018). Using flow cytometry to quantitate macrophage abundance, we found no change in macrophage number 12 h after a session of exercise in this obese cohort. However, we cannot exclude the possibility that the exercise-induced change in adipose tissue macrophage number is transient and was not measured in this time frame. It is also possible that acute exercise does not change macrophage number but may modify the macrophage phenotype instead, consistent with the gene expression-level changes observed by Fabre et al. (2018). In an attempt to stratify macrophage subtypes, we used surface markers CD11c and CD206, but a limitation of our study was that we were unable to achieve consistent separation of macrophage subtypes between cytometer runs. As well as being unable to quantify macrophage subtypes, we observed that most macrophages included using our gating strategy were CD11c^+^CD206^+^, in contrast with previous studies assessing human subcutaneous adipose tissue macrophages (e.g., Wentworth et al., 2010); therefore, further clarification of the response of different macrophage subtypes to exercise is needed.

To assess the ability of exercise to signal cellular remodeling of the broader adipose tissue immune environment, we also quantified DC and T cell content. While T cells (in particular T-regulatory cells and Th2 cells) are important for maintaining adipose tissue health in lean subjects, T cell accumulation in expanding adipose tissue (i.e., weight gain/obesity) reportedly precedes macrophage infiltration (Kintscher et al., 2008; Nishimura et al., 2009), suggesting these cells are important players in early adipose tissue immune responses. Furthermore, CD11c^+^ adipose tissue DCs have been linked to immune cell recruitment and T cell phenotype (Th17 vs. Th1 polarization; Bertola et al., 2012). Here, we found that total T cell and DC numbers did not change 12 h after exercise. Again, this does not preclude the possibility that changes may have occurred earlier or later than 12 h after the exercise session or that exercise induces a phenotypic shift within these cell populations. We also acknowledge that, due to inherent variability of measurements of immune cell content in adipose tissue samples, it is possible that we are underpowered to detect differences in these cell-types in our cohort of obese adults. However, our statistical analyses did not suggest trends for differences in these factors (*p* = 0.2–0.9).

Previous work from our lab (Van Pelt et al., 2017) and others (Walton et al., 2015) suggests that exercise augments subcutaneous adipose tissue angiogenesis, a process required for effective adipose tissue remodeling. Because ECs are central to the process of angiogenesis, we hypothesized that exercise would increase the abundance of these cells in adipose tissue. Contrary to this hypothesis, we did not detect a measurable increase in EC number in adipose tissue collected 12 h after exercise. However, based on evidence that 12 weeks of exercise training increased adipose tissue capillarization (Walton et al., 2015), along with cross-sectional findings from our lab reporting elevated expression of CD31 in regular exercisers compared with sedentary control subjects, we contend it may take longer (many repeated exercise sessions) to detect changes in EC number.

Limitations of this study include lack of power to explore sex differences. While differences in adipose tissue metabolism are known to exist between men and women, we anticipate that these sex-related differences may be subtle compared with the robust effects of exercise on our measures. In addition, because we were interested in studying exercise-mediated signals within *subcutaneous* adipose tissue, our findings may not be translatable to responses in other adipose tissue depots (e.g., visceral or gluteofemoral adipose tissue). While our experiments do not identify a specific mechanism for the reduction in preadipocytes, the preferential reduction in CD34^hi^ preadipocytes suggests that future work could explore these three preadipocyte subtypes (CD34^hi^, CD34^lo^, and CD34^−^) to measure transcriptional/function changes related to differentiation or proliferation. This may be important in the quest to understand how exercise regulates preadipocyte function, as well as to identify pathways for the independent regulation of these progenitor subtypes.

The most novel finding from this study was that exercise decreases preadipocyte number, through a reduction in the abundance of CD34^hi^ preadipocytes, which differentiate into mature adipocytes with high lipolytic rates. Given that high adipose tissue lipolytic rate is linked to insulin resistance (Santomauro et al., 1999; Van Pelt et al., 2014), this phenotypic remodeling could lead to improved adipose tissue and whole body metabolism. Our finding that there was no change in immune cell, macrophage, T cell, or DC content in this cohort suggests that immune cell content is not changed 12 h after exercise in obese regular exercisers. Furthermore, although exercise has been found to increase adipose tissue capillarization (Walton et al., 2015), the lack of change in EC content 12 h after the endurance exercise session suggests that several repeated exercise sessions may be required to detect a measurable increase in angiogenesis. Overall, these data suggest that the rapid effects of exercise on adipose tissue stromal cell number feature a remodeling of the preadipocyte pool that could lead to lower adipose tissue lipolytic rate, thereby improving tissue and systemic insulin sensitivity.

## Data Availability Statement

The raw data supporting the conclusions of this article will be made available by the authors, without undue reservation.

## Ethics Statement

The studies involving human participants were reviewed and approved by University of Michigan Institutional Review Board. The patients/participants provided their written informed consent to participate in this study.

## Author Contributions

AL, LM, CL, and JH conceived and designed the study. AL, EK, TB, MS, CP, BR, LM, KS, CL, and JH analyzed and interpreted the data. AL and JH drafted and critically revised the manuscript. All authors gave approval to all aspects of the final product. All authors contributed to the article and approved the submitted version.

### Conflict of Interest

The authors declare that the research was conducted in the absence of any commercial or financial relationships that could be construed as a potential conflict of interest.
