# Supplementary material for: Acute Aerobic Exercise Remodels the Adipose Tissue Progenitor Cell Phenotype in Obese Adults
Source: Front Physiol. 2020 Jul 28;11:903. doi: 10.3389/fphys.2020.00903 (PMC7399179; doi:10.3389/fphys.2020.00903)
Supplement: Supplementary file 1 [file Data_Sheet_1.DOCX]

Supplementary Material

## Supplementary Figures and Tables

##
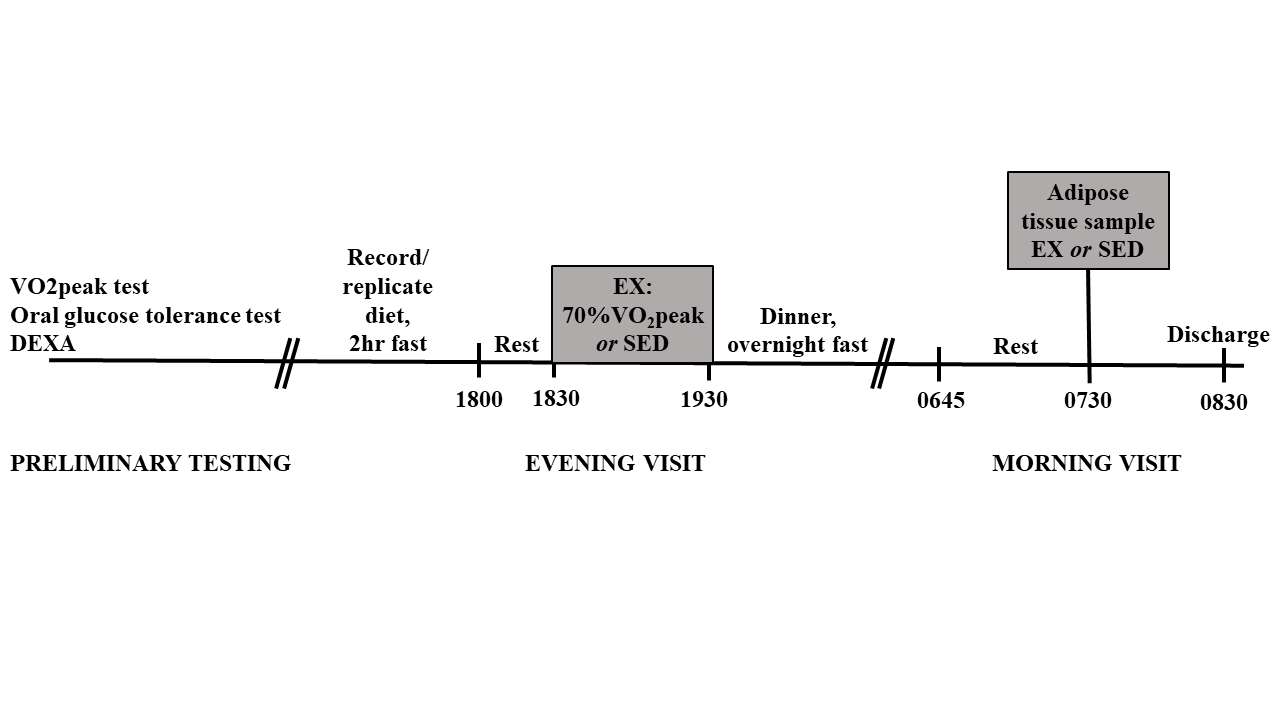


## Supplementary Figure 1.Experimental Design.All participants completed an evening and morning visit for both exercise and sedentary control collections, in randomized order. DEXA: dual-energy X-ray absorptiometry.

##
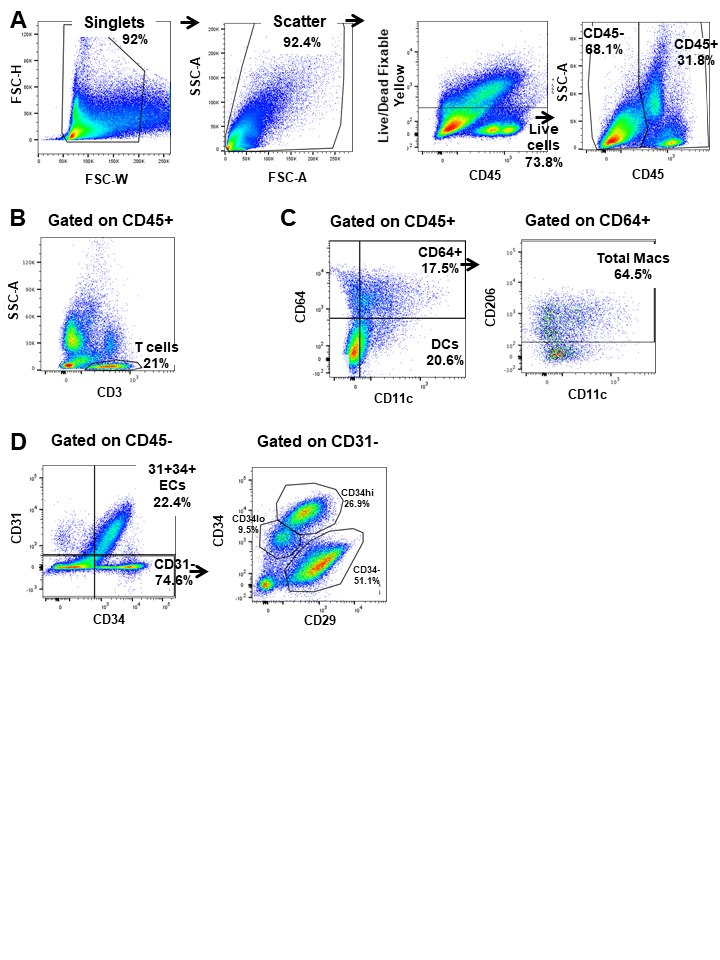


**Supplementary Figure 2. Representative flow cytometry gating scheme.**A) CD45+/- cell populations were gated off of single cells🡪scatter🡪live cell selection. *From CD45+ (immune cells)*: CD3+ T cell selection is shown (2B); as well as total macrophages, CD64+ with the addition of a subsequent CD206- exclusion (2C). *From CD45- (non-immune cells)*: EC’s were selected as CD31+CD34+. Preadipocyte subtypes were gated off of the CD31- selection, based on CD34 and CD29 expression (2D). DC: dendritic cells; EC: endothelial cells; Macs: macrophages.


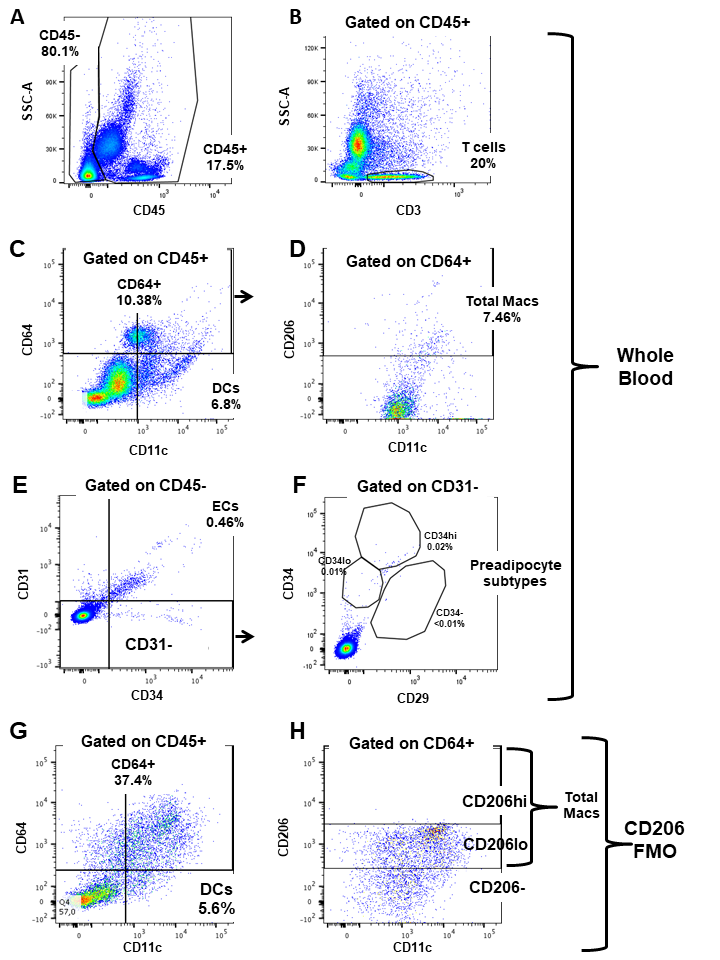


Supplementary Figure 3. Assessment of blood contamination in SVC quantification.(A-F) Whole blood control to assess the presence of blood cells within tissue SVC gating. (G-H) CD206-based selection of a total macrophage population clean from CD64^+^blood cells (blood sample with same gating in 6D). All plots were gated off of singlets, scatter, and live cell gating similar to Supplementary 2.

## TABLES

Supplementary Table - Flow cytometry antibodies

| **Marker** | **Color** | **Clone** | **Supplier** |
| --- | --- | --- | --- |
| Live/Dead | Live/Dead Fix Yellow | N/A | Thermo Fisher Scientific L34967 |
| CD45 | e450 | HI30 | eBiosciences 48-0459-41 |
| CD64 | APC | 10.1.1 | BioLegend 305013 |
| CD34 | Percp-Cy5.5 | 581 | BioLegend 353521 |
| CD31 | APC-Cy7 | WM59 | BioLegend 303119 |
| CD11c | PE-Cy7 | 3.9 | BioLegend 301608 |
| CD29 | PE | TS2/16 | BioLegend 303004 |
| CD3 | FITC | OKT3 | BioLegend 317305 |
| CD206 | BV650 | 19.2 | BD Optibuild 740598 |
| Trustain human FC block | - | - | Biolegend 422301 |
